# Research Progress in Application of 2D Materials in Liquid-Phase Lubrication System

**DOI:** 10.3390/ma11081314

**Published:** 2018-07-30

**Authors:** Lincong Liu, Ming Zhou, Xiao Li, Long Jin, Guoshi Su, Youtang Mo, Liangchuan Li, Hongwei Zhu, Yu Tian

**Affiliations:** 1 School of Mechanical Engineering, Guangxi University of Science and Technology, Liuzhou 545006, China; liulincong123@gmail.com (L.L.); jlong1719@gmail.com (L.J.); suguoshi3@gmail.com (G.S.); youtangmo946@gmail.com (Y.M.); liangchuanli556@gmail.com (L.L.); 2 Chengdu Carbon Co., Ltd., No.88 South2 Road, Economic and Technological Development Zone, Chengdu 610100, China; x-li04@mails.tsinghua.edu.cn; 3 State Key Laboratory of New Ceramics and Fine Processing, School of Materials Science and Engineering, Tsinghua University, Beijing 100084, China; hongweizhu@tsinghua.edu.cn; 4 State Key Laboratory of Tribology, School of Mechanical Engineering, Tsinghua University, Beijing 100084, China; Tianyu@tsinghua.edu.cn

**Keywords:** 2D material, graphene, liquid-phase, lubrication, anti-friction

## Abstract

Two-dimensional (2D) materials are ultra-thin crystals with layered structures that have a monolayer and multiple layers of atomic thickness. Due to excellent performance, 2D materials represented by graphene have caused great interest from researchers in various fields, such as nano-electronics, sensors, solar cells, composite materials, and so on. In recent years, when graphite was used for liquid phase lubrication, there have been many disadvantages limiting its lubrication properties, such as stable dispersion, fluidity and so on. Therefore, 2D materials have been used as high-performance liquid-phase lubricant additives, which become a perfect entry point for high-performance nano-lubricants and lubrication applications. This review describes the application of 2D materials as additives in the field of liquid-phase lubrication (such as lubricating oil and water lubrication) in terms of experimental content, lubrication performance, and lubrication mechanism. Finally, the challenges and prospects of 2D materials in the lubrication field were also proposed.

## 1. Introduction

An estimated one-half to one-third of the world’s energy is consumed by friction and wear. Lubrication aims to effectively reduce friction, wear and energy consumption [1,2]. However, conventional lubricants are limited by their service life and dependence on contact conditions [3]. Therefore, scholars have continuously sought for high-performance and environmentally friendly lubricants [4]. In recent years, carbon nanomaterials, such as fullerenes and carbon nanotubes, have been adopted as additives to water and lubricating oils, but have not been widely used in the friction industry because of their high cost [5].

Graphene was discovered in 2004 by physicists Andre Geim and Konstantin Novoselov [6]. Graphene is a new type of carbon nanomaterial with a hexagonal honeycomb structure composed of single-layer carbon atoms. The thickness of monolayer graphene is only 0.335 nm, which is the thinnest nanomaterial in the world [7]. Its mechanical strength, thermal conductivity, conductivity, and other properties are excellent [6,8]. Two-dimensional materials, such as boron nitride (BN) and molybdenum disulfide (MoS_2_), were also discovered subsequently [9]. Given their excellent performance, 2D materials hold great application potential in various fields including microelectronic devices, sensors, catalysts, batteries, biomedicine, and composite materials [10,11,12,13,14,15,16,17,18,19,20,21,22,23]. Scholars found that the phenomenon of superlubricity exists in layered 2D materials, such as MoS_2_ and BN [24,25,26,27,28,29], and they experimentally investigated the excellent anti-friction properties of 2D materials [30,31,32,33,34,35,36]. Hence, further studies showed that 2D materials can easily enter friction surfaces because of the ultra-thin layer structure and extremely low shear strength between the layers; these characteristics help prevent the direct contact of the friction surface, and decrease the coefficient of friction [37,38,39,40,41,42]. At present, the lubrication mechanisms of 2D materials are mainly as follows [43].

**Figure 1:**
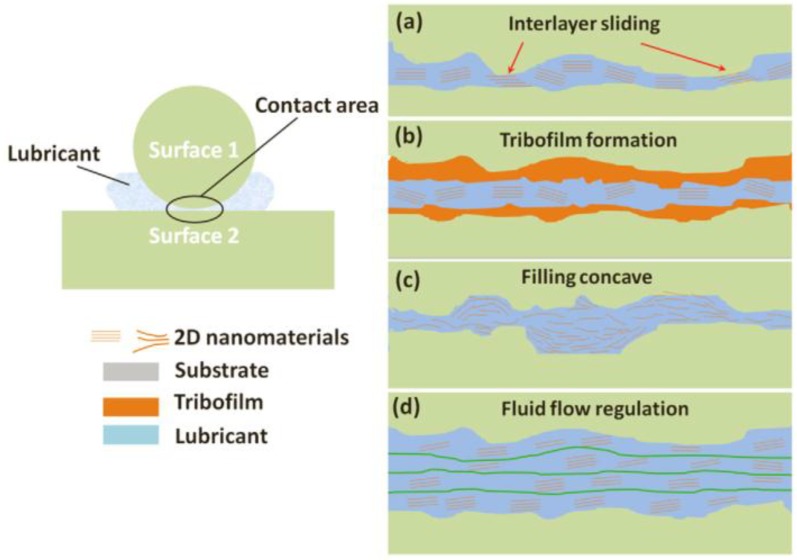
Anti-friction and anti-wear mechanisms of 2D materials. (**a**) entering the contact area of sliding surfaces, (**b**) tribofilm forming, (**c**) filling the pits and gaps of contact area, (**d**) affecting the fluid drag and viscosity. Reproduced with permission from reference Xiao et al. [39], Copyright 2017, Elsevier.

**Figure 2:**
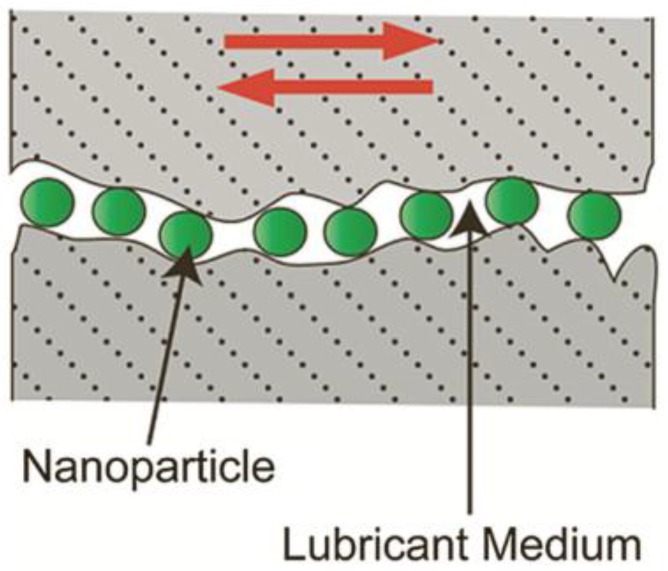
Illustration of a ball bearing mechanism. Reproduced with permission from reference Gulzar et al. [44], Copyright 2016, Springer.

1. Film formation mechanism. On the one hand, 2D materials become quickly adsorbed on a friction surface to form a physical adsorption film, or deposited on a friction surface to form a deposited film. On the other hand, they can also react chemically on the friction surface to create a chemical reaction film, thereby enhancing the wear resistance of the friction pair surface.
2. Self-healing mechanism. Two-dimensional materials can fill a concave area on a friction surface to smoothen it (Figure 1). Gulzar et al. [44] investigated the self-healing effect of nanomaterials. Nanoparticles deposit on interacting surfaces and compensate for the mass lost, thereby reducing wear and tear.
3. Ball bearing mechanism. Two-dimensional materials disperse at the contact surface to form ‘class bearings’ and transform sliding friction into rolling friction, thereby showing excellent anti-friction performance (Figure 2) [44].

Two-dimensional materials not only exhibit excellent anti-friction and anti-wear properties, but also have reasonable preparation costs in the field of lubrication. Thus, 2D materials are selected as high-performance and environmentally friendly lubricants [45,46,47,48,49,50,51,52,53,54,55,56,57,58,59,60].

Liquid-phase lubrication is a type of lubrication system with many varieties in the industrial field. This type of lubrication system is effective for high-speed and high-load conditions in the industry because of the advantages of forming a special protective film, low friction, and low energy consumption. Liquid-phase lubrication can generally be divided into oil-based lubrication and water-based lubrication, and it can convert external friction between two moving surfaces into internal friction between protective films, thereby separating the two moving surfaces and reducing friction. In general, liquid-phase lubrication is the most widely used in traditional industrial fields such as construction machinery, steel, and automobiles. The advantages are that not only can the friction and heat of the equipment be reduced, but also its safety and reliability can be improved. 

Graphite is a common standard for lubrication. However, when used in liquid-phase solution, there are many disadvantages limiting its lubrication properties. First, it is hardly possible to disperse steadily in liquid-phase solution. Second, it weakens the fluidity and reduces the lubrication performance of lubricants. Third, graphite is barely deposited on the rubbing surface, which confirms that graphite has difficulty entering the contact area and, consequently, forming a continuous protective film. By contrast, graphene can be stabilized by being modified with a surfactant or by chemical modification. Graphene easily forms protective deposited films to prevent the rubbing surfaces from coming into direct contact and thereby improves the entirely tribological behavior of the oil. Finally, during the sliding process, graphene with higher exfoliation easily forms to lamellar a protective film [61]. Thus, graphene can be transported into the friction zone by the liquid flow. Two-dimensional materials can be used in a liquid-phase lubrication system. For instance, Song et al. [62] investigated the lubricating properties of graphene oxide (GO) as a water-based lubricant additive. Under a load of 60 N, the water-lubricated friction coefficient of GO with a mass fraction of 1% was 0.127. GO can easily enter the two contact surfaces and form a friction film on the contact surface to prevent direct contact of the steel balls. Chen et al. [63] synthesized oil-soluble ultra-thin MoS_2_ sheets by the solvothermal method. These sheets effectively control the wear when the rated load reaches about 1 GPa. The mechanism of lubrication involves ultra-thin MoS_2_ sheets easily entering and adhering to the contact surface; such adherence helps smoothen the sliding interface. In addition, different 2D materials possess unique characteristics. For example, boron nitride maintains good lubricating properties at high temperatures [64]. Nanometer-scale ultra-thin MoS_2_ can greatly improve the extreme pressure performance of lubricating oil [65]. Therefore, the application of 2D materials as additives for liquid-phase lubrication can further increase the effective performance by improving the lubrication characteristics, which hold a broad prospect.

This review describes in detail the research results and advances in the use of 2D materials as additives to liquid-phase lubrication, such as lubricating oil and water lubrication systems, in terms of experimental content and lubricating properties, lubrication mechanism and influencing factors in recent years. Finally, the review summarizes the prospects and challenges amongst 2D materials in this field and proposes a series of feasible measures.

## 2. Field of Lubricating Oil

Two-dimensional materials can significantly reduce the friction coefficient and achieve a highly efficient lubricant. However, 2D materials must be stably dispersed when utilized in lubricating oil [66,67,68]. The fully structured 2D materials exhibit high chemical stability and van der Waals forces between layers and are thus prone to irreversible agglomeration. Physical and chemical methods can be used to improve the dispersion of 2D materials in base oil. Studies on 2D materials-based additives to lubricating oil are described in the following sections.

### 2.1. Two-dimensional Material Lubrication Performance and Mechanism

**Figure 3:**
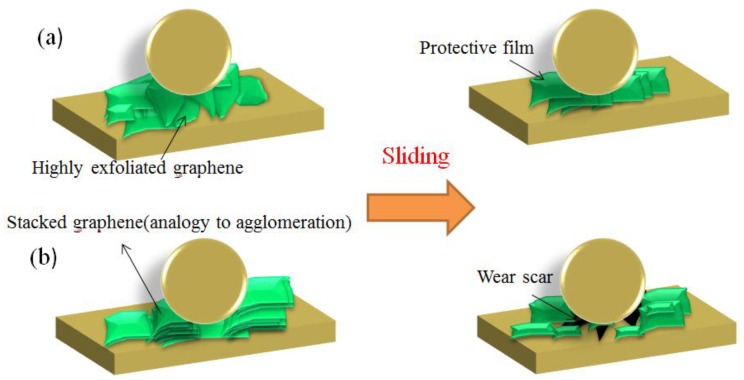
The lubrication mechanisms of graphene in different degrees of peeling. (**a**) During the sliding process, graphene with higher exfoliation restacks to lamellar a protective film. (**b**) The stacked graphene layers are prone to being damaged, and further appear as a wear scar. Reproduced with permission from reference Zhao et al. [73], Copyright 2018, Elsevior.

As a new type of lubricating additive, most 2D materials not only provide excellent anti-friction and anti-wear properties, but are also green additives [69]. Chen et al. [70] explored the lubricating properties and anti-friction mechanisms of few-layer GO sheets as hydrocarbon-based lubricating oil additives. By comparing the wear scars at different concentrations, it has been found that the concentration of the GO sheet gave the best performance at 0.5%. In addition, GO provided lubrication by acting as a protective film to prevent the direct contact of the rugged surface, and hence reduced friction and wear. With the advancement of technology, simple routes for synthesizing high-performance graphene have been developed. When used as additives, 2D materials exhibit great potential for industrialization [71]. Zhao et al. [72] prepared GO by thermal reduction at 700 °C. Compared with base oil without graphene, base oil with GO achieved a friction coefficient that can be reduced by as much as 30%. Graphene holds the advantages of low-cost, simple operation and massive potential application. Zhao et al. [73] studied the lubricating properties of graphene with different layers and space by investigating the degree of peeling of graphene. Experiments showed that lubricating oil with added few-layer graphene of large interlayer space, decreased the friction coefficient and shrank the wear marks. The ordered friction film on the friction interface was parallel to the sliding direction. This observation indicated that few-layer graphene with large interlayer space can effectively improve the lubricating property of the oil by forming an ordered friction film at the friction interface. To accurately describe the friction mechanism, they constructed a theoretical sketch (Figure 3). Graphene additives with a higher degree of exfoliation underwent ordered deposition changes during the friction process, which resulted in enhanced lubricating properties. Besides graphene, other 2D materials also function as additives in lubricating oils [74,75,76]. Zhang et al. [77] prepared ultra-thin WS_2_ nanosheets using a simple solid-state reaction. The UMT-2 disc friction wear tests showed that the anti-friction and anti-wear properties were best when the concentration of WS_2_ was 1%. They attributed this loss to the addition of ultra-thin WS_2_ nanoplatelets, which enter the friction interface and form a continuous friction film on the friction surface. Chen et al. [78] reduced spherical MoS_2_ under hydrogen by using ultrasonic chemistry. When added to lubricating oil as an additive, the lubrication performance of the lubricant was significantly improved. The authors summarized that MoS_2_ nanoparticles can significantly improve the lubricating properties of lubricating oils, which were attributed to the lubrication mechanisms of surface repair, rolling friction effect and film lubrication. Bai et al. [79] prepared MoS_2_ nanospheres by using a surfactant-promoting method. They also concluded that MoS_2_ nanospheres significantly enhance the lubricating properties of oils. On one hand, the nanospheres were easily adsorbed onto the contact surface and effectively filled the wear scar on the surface to form a lubricating protective film because of the smaller particle size and higher surface activity, which can achieve a lower friction factor over a prolonged period of time. On the other hand, this result is attributed to “the compact friction” and “sliding friction” compound friction mechanisms. In a word, using 2D materials as lubricating additives can significantly improve the lubricating properties of lubricating oils.

### 2.2. Modification of 2D Materials 

**Figure 4:**
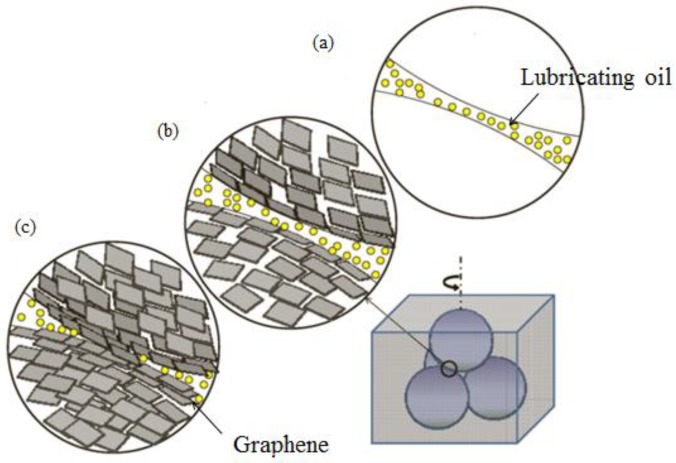
Schematic of the four-ball friction test. (**a**) Friction diagram of pure lubricating oil. (**b**) Represents optimal concentration of graphene in oil. (**c**) Represents excessive concentration of graphene in oil. Reproduced with permission from reference Zhang et al. [83], Copyright 2011, IOP.

Although 2D materials possess excellent lubricating properties, they can easily cause irreversible agglomeration in the lubricating oil because of their large specific surface area. Two-dimensional materials must be modified to enhance the lubricating performance [80,81,82]. Lin et al. [61] found that graphene sheets modified with stearic acid and oleic acid can be uniformly dispersed in lubricating oils. When the mass fraction was 0.075%, the anti-wear loading capacity was best. This effect was achieved because the long paraffin chains on the surface of graphene produce a steric hindrance to prevent graphene sheets from being precipitated and agglomerated when modified graphene is dispersed in base lubricating oil. Zhang et al. [83] utilized the modification effect of an oleic acid to disperse the liquid-phase exfoliated graphene sheet uniformly in poly-α-olefin (PAO9) lubricating oil, and used a four-ball abrasion tester to assess the lubrication performance of modified graphene (Figure 4). As a result, the friction coefficient and wear spot diameter decreased by 17% and 14%, respectively. Therefore, the modified graphene exhibited an excellent lubricating performance. The conclusion is consistent with Chen et al. [84]. Although hexagonal boron nitride (h-BN) possesses a layered structure similar to that of graphene, and a high thermal stability and oxidation resistance, the poor dispersion of h-BN in lubricating oil seriously affects lubrication applications. Kumari et al. [85] prepared modified h-BN by oxidizing h-BN nanosheets to produce hydroxyl functional groups and chemically covalently grafting long alkyl chains. The van der Waals interaction between the alkyl chain in the h-BN and the hydrocarbon groups in the lubricating oil promoted long-term dispersion stability. Fully dispersed modified h-BN significantly improved the lubricity of the lubricant, which was attributed to the low shear resistance provided by the lamellar structure. Elemental mapping on the track of the wear scar shows that a h-BN film was formed on the contact interface, which offered protection. MoS_2_ is easily sheared at the contact interface because of the weak interaction between layers, which can be used as an excellent lubricant additive. Kumari et al. [86] utilized a simple and scalable hydrothermal method to synthesize MoS_2_ nanosheets, and covalently grafted long alkyl chains for modification. The van der Waals force interaction between the alkyl chain and the lubricating oil was used to stably disperse MoS_2_ in the lubricating oil for a long time, and the weak van der Waals force and the easy shearing properties between the formed friction films enhanced the lubricating performance. Li et al. [87] prepared nanometer MoS_2_ lubricating oil with oleic acid surfactant which was dispersed by ultrasonic treatment. This action not only enhanced the dispersion of the MoS_2_ in lubricating oil, but also the anti-wear performance of 0.01% MoS_2_ in lubricating oil and the anti-wear performance under high load.

### 2.3. Compound Treatment of 2D Materials 

**Figure 5:**
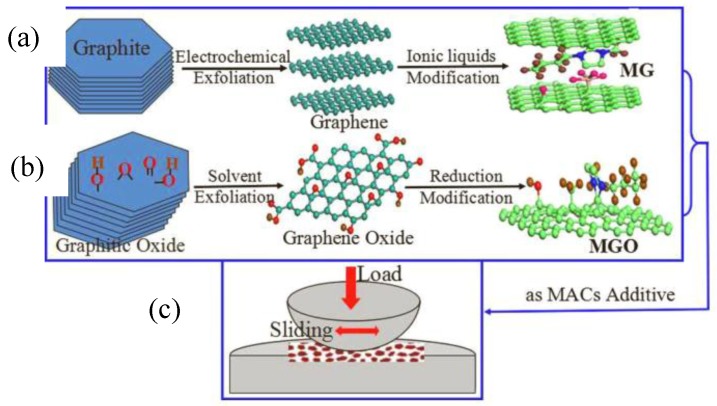
The preparation and tribological properties tests of modified graphene (MG) and modified graphene oxide (MGO). (**a**,**b**) Modified graphene (MG) and modified graphene oxide (MGO) were prepared using alkyl imidazolium ionic liquids by electrochemical exfoliation and solvent exfoliation, respectively. (**c**) Schematic of tribological properties tests of MGO and MG. Reproduced with permission from reference Fan et al. [92], Copyright 2015, Elsevior.

**Figure 6:**
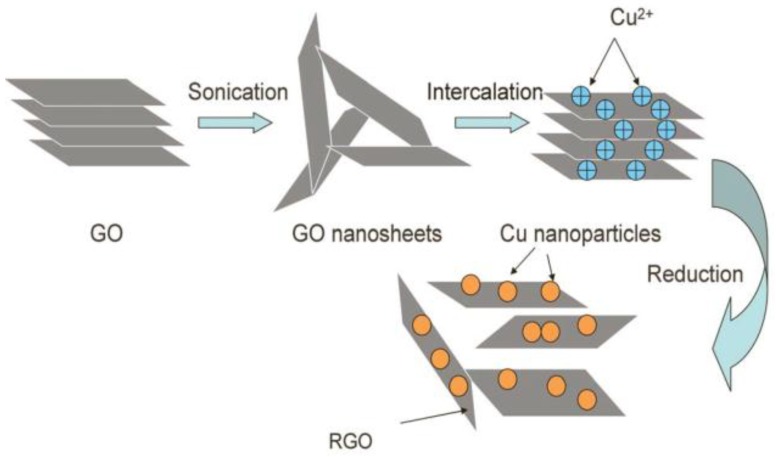
Preparation of RGO/Cu nanoparticle composites. Step 1: Graphene oxide (GO) nanosheets are exfoliated to lots of single GO nanosheets and the metal cations are inserted on single GO nanosheets.Step 2: Cu nanoparticles are generated on the action of strong reductant. Reproduced with permission from reference Zhang et al. [98], Copyright 2013, RSC Advances.

The compounding of 2D materials can produce synergistic effects, which not only stabilize the dispersion, but also improve the lubrication performance [88,89,90,91,92,93] (Figure 5). Gusain et al. [94] synthesized graphene-ionic liquid (Gr-IL) hybrid nanomaterials by reducing GO and covalently grafting imidazole rings. Elemental analysis of the wear traces of the sample showed that the friction chemical film was formed during the lubrication process of the hybrid nanomaterial, and the carbonaceous/graphene friction belt played a key role in reducing the friction. Graphene also significantly improved the wear resistance while covalently grafting ionic liquids to promote complete dispersion in lubricating oils. Therefore, Gr-IL hybrid nanomaterials play a synergistic role in dispersion and friction reduction as a new type of lubricating additive; the nanomaterials are excellent candidate modern lubricating additives. Zhang et al. [95] tested the tribological properties of 2D layered molybdenum disulfide additive ionic liquid (IL-MoS_2_) by using a vacuum friction tester. Studies have shown that in the friction process, the ionic liquid and the 2D layered molybdenum disulfide act synergistically to produce a frictional chemical reaction on the surface of stainless steel to form a stable chemical reaction film, which exerts excellent anti-friction and anti-wear effects on steel-steel frictional energy. Jing et al. [96] prepared molybdenum dialkyldithiocarbamate (MoDTC) and graphene composite lubricating oil additives and used an Optimal-SRV4 friction and wear testing machine for lubrication performance testing. The composite additive generated a stable adsorption film and chemical reaction film on the friction surface under high temperature and high load, and hence exhibited an excellent lubricating effect. Upon compounding, graphene and MoDTC exhibited a synergy of friction reduction and significantly improved the lubricating performance of MoDTC under high temperature. As such, the materials suggest great prospects. Feng et al. [97] modified nano-Cu particles and graphene with oleic acid and octadecylamine, which can be uniformly and stably dispersed in lubricating oil. The modified nano-Cu particles and graphene were compounded at different ratios and tested using a vertical universal friction and wear tester. Results showed that when the mass ratio WtCu%: WtGr% was 1:4, the friction coefficient and wear scar diameter were reduced by 31.43% and 22.31%, respectively. Zhang et al. [98] synthesized an reduced graphene oxide (RGO) /Cu nanoparticle composite by a chemical reduction method (Figure 6) and studied the lubricating properties of the material as a lubricant additive by friction tester. Compared with lubricating oils containing only RGO nanosheets, the composites exhibited superior wear resistance, load carrying capacity, and lubricating properties, which were attributed to the synergistic mechanism of them in base oils.

Although 2D materials show excellent anti-friction and anti-wear properties as additives in lubricating oils, factors such as process methods, mass fractions, and newly generated materials in the lubrication process significantly impact the performance. Zhang et al. [99] revealed that adding graphene at an excessively high concentration increases the friction and wear. Gupta et al. [100] found that the use of RGO as an additive to lubricating oils was the main reason that the friction and wear of lubricated solid contacts was controlled. The low shear strength of the sheet allowed easy sliding and provided effective lubrication. Deepika et al. [101] prepared BN nanosheets through wet ball milling, which did not cause damage to the planar structure, and reduced the wear traces and friction coefficients of the base oil in the four-ball test. As a result, the lubricity was significantly enhanced. Method optimization greatly influences material properties and holds a guiding significance. Song et al. [102] demonstrated the excellent anti-friction effect of graphene after analyzing the influence of graphene on the anti-friction performance of engine oil, hydraulic guideway oil and grease. However, the resulting oxide was easily combined with graphene and filtered by the lubrication system, and hence affected the application of graphene. This result indicated the key issues in using graphene in greases. Therefore, further study is needed to optimize the amount of graphene in lubricating oil and the lubrication mechanism of graphene.

## 3. Field of Water Lubrication 

Water lubrication holds the advantages of environmental protection, energy conservation and excellent heat conduction properties. Adding a good water-lubricant additive can significantly improve the water-lubricant performance [103,104,105,106]. At present, the excellent lubricating properties of 2D materials as additives in the field of water lubrication has attracted great interest [107,108,109,110,111,112]. The studies on using 2D materials in the field of lubricating oil are described in the following sections.

### 3.1. 2D Material Lubrication Performance and Mechanism

**Figure 7:**
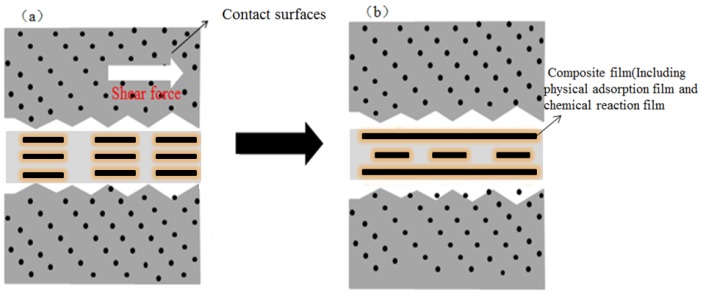
Schematic of the water lubrication mechanism of liquid graphene. (**a**) The individually dispersed graphene sheet on the contact surface; (**b**) during the sliding process, the individually dispersed graphene sheets can be rearranged on the surface to form a composite protective film, which effectively reduces friction and wear. Reproduced with permission from reference Yang et al. [115], Copyright 2017, Elsevier.

Liu et al. [113] examined the lubricating properties of GO and modified diamond nanoparticles as water-lubricant additives. By adding GO nanosheets, the friction coefficient decreased sharply from 0.6 to 0.1, and a steady state of ultra-low friction (0.01) was finally obtained. Concurrently, the running time of the friction pair was shortened from 2000 s when pure water was lubricated to 250 s. For modified diamond nanoparticles, the coefficient of friction was maintained at 0.1. They attributed the excellent performance of GO to its layered structure and geometry, and pointed out that GO nanoplatelets are the most promising “candidate” water-based lubricant additives. Elomaa et al. [114] studied the lubricating properties of 0–2 wt % GO dispersed in water. When a normal load of 1 wt % GO and 10 N was used, the friction coefficient was reduced by 57% relative to that of pure water. As the GO concentration increased from 0 to 1 wt %, the coefficient of friction exhibited a continuous decrease from 0.14 to 0.06. Therefore, at the optimized GO concentration of 1 wt % (theoretical value), the graphene concentration for optimal performance can be adjusted. The authors analyzed that the lubrication mechanism can be attributed to the insertion of GO into the contact surface of steel and the binding of water molecules on GO, and GO was superior to graphene in water lubrication. Yang et al. [115] successfully grafted an amino-terminated block copolymer (M2070) onto the surface of graphene by surface sulphonation, neutralization and ion bonding. At room temperature, the modified graphene showed a liquid-like behavior, which rendered the graphene well dispersed in aqueous solution. When the concentration of the liquid-like graphene was 50 mg/mL, a reduction in the friction coefficient and wear rate occurred by 53% and 91%, respectively. For the lubrication mechanism (Figure 7), the liquid graphene possessed an ion-bonded core-shell structure and the metal surface carried a positive charge during the sliding process. Therefore, the graphene with a negative charge adsorbed onto the surfaces of the sliding pair because of electrostatic adsorption. The M2070 molecules then rearranged on the surface of the graphene to form a composite friction film, and effectively reduced the friction. This process is similar to that of graphene with liquid-like behavior modified by Tang et al. [116].

**Figure 8:**
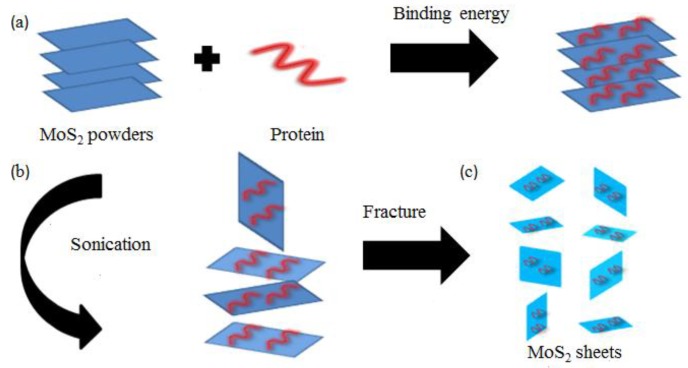
Schematic of protein-induced super-thin MoS_2_ flakes. (**a**) Step 1: Protein are strongly adsorbed on MoS_2_ surfaces via binding energy. (**b**) Step 2: MoS_2_ flakes are exfoliated via ultra-sonication. (**c**) Step 3: Layer-by-layer fracture of MoS_2_ flakes takes place gradually. Reproduced with permission from reference Liu et al. [121], Copyright 2016, RSC Advances.

Besides graphene, other 2D materials show excellent anti-friction and anti-wear properties in the field of water lubrication [117,118]. Cho et al. [119] synthesized an aqueous dispersion of h-BN nanosheets without using any dispersant. Optical absorbance data showed that the h-BN dispersion can be stably dispersed in water for 30 days, and even a small amount of h-BN nanoplatelets can enhance the wear resistance and reduce the friction coefficient. This result was achieved because a friction film that can reduce friction and wear was formed on the sliding surface. The simply synthesized and stably dispersed h-BN nanoplatelets can be a promising “green” lubricant additive to water. He et al. [120] studied the effect of α-zirconium phosphate on the lubrication of 2D nanomaterials. The characterization technique revealed that when the nanomaterial sheets were used as a lubricating oil additive into oily and aqueous media, the friction coefficient was reduced by 65% and 91%, respectively. MoS_2_ with good dispersibility can also be prepared using a protein, which is highly suitable as additive to water lubricants. Liu et al. [121] prepared super-thin MoS_2_ nanoplates by ultrasound treatment under the induction of bovine serum albumin (BSA) or hemoglobin (HB) (Figure 8). BSA-MoS_2_ and HB-MoS_2_ exhibited good dispersion and stability in pure water, and also formed protective films on friction surfaces. When each additive content was 0.1 wt %, the average friction coefficient was stable at 0.06 or 0.08, respectively. This process can realize cross-domain applications and holds great scientific value.

### 3.2. Compound Treatment of 2D Materials

Two-dimensional material can be compounded with other materials to improve water lubrication performance [122,123,124]. Hou et al. [125] prepared LaF_3_-GO nanohybrids by a simple solution method and analyzed their lubricating performance as a distilled water additive through a four-ball friction wear tester. Results showed that the lubricating properties of distilled water can be significantly enhanced by adding LaF_3_-GO nanohybrids at a mass fraction of 1.5%. The superb performance was attributed to the deposition of LaF_3_-GO nanohybrids onto the friction surface under water lubrication; the nanohybrids formed a protective layer and a lubricating layer composed of GO, LaF_3_, Fe_2_O_3_, and FeF_3_, and thereby significantly diminished the friction and wear of the steel-steel contact. Qiao et al. [126] prepared graphene-nano Fe_3_O_4_ composites by using a liquid phase lift-off method. The friction coefficient and the wear volume of the graphene-nano Fe_3_O_4_ composites of 0.01 wt % concentration decreased by 26.7% and 35.4%, respectively. The lubricating mechanism also involved the formation of an adsorption film, boundary lubricating film containing graphene and nano-Fe_3_O_4_ on the worn surface, which significantly reduced the friction and wear.

Although graphene plays an excellent anti-friction and anti-wear performance in water lubrication, the pH of the aqueous solution seriously affects the performance of graphene lubrication. Alias et al. [127] investigated the lubricating properties of graphene dispersed in distilled water at different pH values by using SUS304 steel plates and tungsten carbide (WC) steel balls. When the pH was 3, the friction coefficient between the SUS304 steel plate and the WC steel ball was 0.05. As the pH increased, the friction coefficient and wear width of the steel plate and steel ball also rose. This result was achieved because the friction film was formed at low pH to reduce frictional wear. Finally, the group proposed that the chemical chain of GO can be modified to increase its lubricating properties as a water lubricant additive. He et al. [128] also investigated the effect of pH on the tribological properties of GO suspensions. Compared with pure water, an acidic GO suspension reduced the friction coefficient by 44.4% and the wear radius by 17.1%. An increase in pH also resulted in an increased coefficient of friction and significantly increased wear. The acidic GO suspension lubricates by forming a thin friction film to separate the contact points, and hence diminishes the friction and wear. Further study is needed to examine the factors that affect the lubricating performance of graphene as an additive to water and find the measures for enhancing the lubrication performance.

## 4. Summary and Outlook

Given their unique structures and excellent properties, 2D materials have attracted great attention. In particular, the unique properties such as ultra-thin layers and large specific surface area are highly suitable for nano-lubricant application. This review described in detail the application results of 2D materials such as graphene in liquid-phase lubrication, such as lubricating oil and water lubrication systems. However, current research on the lubrication of 2D materials still faces many challenges and entails further investigation in the following areas:

1. Although modification can effectively solve the dispersion problem, the controllability remains to be tested. Moreover, industrial production has not been realized and merits further exploration.
2. In order to improve the dispersion of 2D materials in a liquid phase environment, more effective modification methods should be explored.
3. Some modifiers not only promote the uniform dispersion of graphene, but also operate synergistically with the material. The working mechanism of such modifiers entails further study, with the aim of development in other fields.
4. Two-dimensional materials exhibit excellent anti-friction and anti-wear performance in the lubricating process, and their lubricating mechanism requires further exploration.
5. Factors such as process methods, concentration changes, and new substances greatly influence the lubricating effect. Therefore, it is necessary to reasonably control the factors affecting the lubrication effect to maximize the lubrication ability of 2D materials.
